# Antimicrobial susceptibility of *Brucella* spp. isolated from Iranian patients during 2016 to 2018

**Published:** 2019-10

**Authors:** Saeed Alamian, Maryam Dadar, Afshar Etemadi, Davoud Afshar, Mohammad Mehdi Alamian

**Affiliations:** 1 Department of Brucellosis, Razi Vaccine and Serum Research Institute (RVSRI), Agricultural Research, Education and Extension Organization (AREEO), Karaj, Iran; 2 Department of Microbiology and Virology, School of Medicine, Zanjan University of Medical Sciences, Zanjan, Iran; 3 Department of Microbiology and Immunology, Tehran Medical Sciences Branch, Islamic Azad University, Tehran, Iran

**Keywords:** *Brucella*, Antimicrobial agents, Brucellosis

## Abstract

**Background and Objectives:**

Brucellosis is a widespread zoonotic disease with a high prevalence in both animals and humans. The present study was aimed to evaluate the susceptibility of *Brucella* strains isolated from human clinical specimens against commonly used antimicrobial agents.

**Materials and Methods:**

A total of 360 blood specimens were collected during 2016–2018 and subjected to culture and *Brucella* spp. identification. The classical biotyping for *Brucella* isolates was performed according to Alton and coworker’s guidelines. Antimicrobials susceptibility test carried out using disk diffusion and minimal inhibitory concentration (MIC) methods.

**Results:**

In this study, sixty *B. melitensis* strains were isolated from blood samples (16%) and all them belonged to biovar 1. Majority of the tested antibacterial agents, excepting ampicillin-sulbactam had an effective activity against *B. melitensis* isolates in E-test (MIC) and disk diffusion method. Moreover, probable resistance to rifampin and ampicillin-sulbactam were observed in 60 (100%), 1 (1.7%), 11 (18.4%) and 2 (3.4%) isolates, respectively.

**Conclusion:**

Our data suggest that the efficacy of commonly used antibiotics for brucellosis treatment should be regularly monitored. In conclusion, appropriate precaution should be exercised in the context of antibiotic administration to prevent future antibiotic resistance.

## INTRODUCTION

Brucellosis is a zoonotic disease that affects both animals and humans, causing significant economic and public health problems in many countries worldwide. *Brucella melitensis* and *Brucella abortus* are two main species that cause epidemic brucellosis in Iran (1, 2). Also, human brucellosis may be caused by different *Brucella* species, i.e., *B. canis* (from dogs) and *B. suis* (from pigs), through infected organs of animals or the consumption of unpasteurized milk and milk products (3). Brucellosis usually causes outstanding economic losses through the reduction in milk yield and animal abortion. Therapeutic strategies in the brucellosis treatment have not been effective absolutely, and disease relapses have been reported in many cases (1). Many studies have also revealed that the inappropriate and widespread use of antibiotics may lead to antibiotic resistance among *Brucella* isolates (2, 4, 5).

It is now well-established that *Brucella* is an intracellular bacterium which escapes from macrophage killing (6), and causes severe mitochondrial fragmentation following 48 hours of bacterial entry into the different cell types (7). Hence, the antibiotics for the brucellosis treatment needs to have the ability to kill bacteria through the penetration into the macrophages (8).

In many clinical laboratories, the antibiotic susceptibility testing is not routinely applicable due to lack of biosafety level 3 facilities (9). Thus, there are some limited data about the antibiotic susceptibility of *Brucella* species and its determination seems to be necessary. Therefore, the present study was aimed to investigate the antibiotic susceptibility profile of *Brucella* spp. by disk diffusion and MIC approaches.

## MATERIALS AND METHODS

### Specimens and culture

A total of 360 blood specimens were collected from patients with brucellosis admitted to Brucellosis department of Razi vaccine and serum research institute in Karaj, Iran from 2016 to 2018. All blood specimens were cultured on *Brucella* selective agar supplemented with bacitracin (12,500 IU), polymyxin B (2,500 IU), cycloheximide (50.0 mg), vancomycin (10.0 mg), nystatin (50,000 IU), nalidixic acid (2.5 mg) (Oxoid, UK) and 5% inactivated horse serum and incubated at 37°C for 10 days under 10% CO_2_ condition. The grown colonies were subcultured on the Brucella-specific agar (Himedia, India) and incubated at 37°C for 7 days. Typical colonies of *Brucella* spp. were subjected to further analysis to define their biotypes (10).

### *Brucella* biotyping

The classical biotyping for *Brucella* isolates was performed according to Alton and coworkers procedure (11). In the present study, a panel of biotyping tests including agglutination by acriflavine, lysis by specific phages (Tbilisi (Tb) and Izatnagar (IZ)), growing in media containing thionin and basic fuchsine, H_2_S production, carbon dioxide (CO_2_) dependence, and agglutination with specific *Brucella* antisera were performed (12) and finaly, their results interpreted according to the OIE manual (http://www.oie.int/en/animal-health-in-the-world/animal-diseases/Brucellosis).

### Antimicrobial susceptibility test

For each isolate, a bacterial suspension was prepared from pure and fresh colonies and the tube turbidity adjusted to the 0.5 McFarland turbidity standard. The suspensions were spread onto Muller-Hinton agar plates supplemented with 5% sheep’s blood and incubated at 37°C in the presence of 10% CO_2_. Minimum Inhibitory Concentration (MICs) of clinical isolates to gentamicin (0.064–1024 μg/ml), streptomycin (0.064–1024 μg/ml), rifampin (0.016–256 μg/ml), doxycycline (0.016–256 μg/ml), ceftriaxone (0.016–256 μg/ml), ampicillin (0.016–256 μg/ml) and trimethoprim/sulfam (0.002–32 μg/ml) were measured using the E-test method (liofilchem/Italy) recommended by the Clinical and Laboratory Standards Institute (CLSI) guidelines. Disk diffusion susceptibility tests were performed for the antibiotics gentamicin (10 μg per disk), streptomycin (10 μg per disk), rifampin (5μg per disk), doxycycline (30 μg per disk), ceftriaxone (30 μg per disk), ampicillin-sulbactam (10 + 10 μg per disk) and trimethoprim/sulfam (1.25/23.75 μg per disk). The results of all antimicrobial tests were read after 48 h.

The breakpoints of *Brucella* against the tested antibiotics have been established according to the guidelines for slow-growing bacteria (*Haemophilus* spp.) as previously reported by other groups. All antibiotics were assessed in duplicate for all isolates (9,13–16).

## RESULTS

A total of 60 *B. melitensis* strains isolated from blood specimens and agglutinated with anti-M monospecific sera, grew in the presence of thionin and basic fuchsine without CO_2_ requirement. Also, all isolates were H_2_S negative and were not affected by the Tb phage. Conversely, the IZ phage exerted lytic activity on all tested isolates. According to our results, all isolates were identified as *B. melitensis* biovar 1, despite the diversity of their geographical origins (54 isolates form Kermanshah, two isolates from Zanjan and one isolate from each of Kerman, Esfahan, Hamedan, and Karaj provinces).

According to MIC measurements, all of the tested isolates appeared to be susceptible to ceftriaxone (MIC90=0.75 μg/ml), doxycycline (MIC90=0.25 μg/ml), streptomycin (MIC90=0.75 μg/ml), trimethoprim-sulfamethoxazole (MIC90=0.19 μg/ml), and gentamicin (MIC90=0.75 μg/ml) (Table 1).

**Table 1:** The MIC values of antibiotics against 54 human *Brucella* isolates

**Antibiotic**	**MIC Range (μg/ml)**	**MIC Range 50 (μg/ml)**	**MIC Range 90 (μg/ml)**	**CLSI Breakpoints for Brucella (μg/ml)**		

				**S**	**I**	**R**
Ceftriaxone	0.12–1	0.250	0.75	≤ 2	-	-
Doxycycline	0.047–0.19	0.064	0.25	≤ 4	8	≥16
Rifampicin	0.125–1.5	0.38	1	≤ 1	2	≥4
Streptomycin	0.38–1	0.94	0.75	≤ 8	-	-
Trimethoprim						
Sulfamethazole	0.016–0.64	0.032	0.19	≤ 0.5	1–2	≥4
Gentamycin	0.094–1.5	0.38	0.75	≤ 4	-	-
Ampicilin sulbactam	0.19–6	1.5	2	≤ 1	2	≥4

Standard breakpoints are from CLSI guidelines for slowly growing bacteria (*Haemophilus* spp.)

The MIC values for rifampin ranged from 0.125–1.5 μg/ml, which is in line with CLSI breakpoints for slow-growing bacteria (*Haemophilus* spp.), although a MICs≥4 μg/ml was also observed in 1 isolate (1.7%) which leads to resistance phenotype, probably. Similarly, only eleven (18.4%) isolates showed possible resistance to ampicillin-sulbactam in disc diffusion tset (Table 2).

**Table 2:** Antibiotic sensitivity of human specimens obtained by disk diffusion test

**Antibiotic**	**Concentration μg/disk**	**Range (mm)**	**Sensitive No (%)**	**Intermediate No (%)**	**Resistant No (%)**	**Antimicrobial sensitivity**		

						**S**	**I**	**R**
Ceftriaxone	CRD30	28–55	60 (100)	0	0	≥26	-	-
Doxycycline	D30	31–48	60 (100)	0	0	10 ≥	-	-
Rifampicin	RA5	16–38	59 (98.4)	0	1 (1.7)	≥20	17 19	≤16
Streptomycin	S10	19–36	60 (100)	0	0	80≥	-	-
Trimethoprim Sulfamethazole	SXT1.25/23.75	23–42	60 (100)	0	0	≥16	11 15	≤10
Gentamicin	GM10	22–45	60 (100)	0	0	≥16	-	-
Ampicilin sulbactam	Sam20	11–32	49 (81.7)	0	11 (18.4)	≥20	-	≤19

## DISCUSSION

Recently, increasing microbial resistance to common antibiotics has attracted much considerations to select new classes of antibiotics for the specific treatment of infectious diseases. According to the World Health Organisation (WHO), only some restricted antibiotics are with clinical efficiency and good intracellular penetration for the brucellosis treatment (14). Our study demonstrated that many *B. melitensis* strains isolated from different regions of Iran were susceptible to a broad panel of antibiotics including gentamicin, streptomycin, rifampin, doxycycline, ceftriaxone, ampicillin and trimethoprim/sulfamethoxazole. Resistance to rifampin was only observed in 1 (1.7%) of the isolates. A study from Iran assessing the pattern of antibiotic susceptibility in 140 clinical isolates of *B. melitensis* from Hamedan (western of Iran) demonstrated that all isolates were sensitive to streptomycin, doxycycline, ciprofloxacin, moxifloxacin, and gentamicin, but intermediate sensitivity to trimethoprim-sulfamethoxazole and rifampin were also found in 3.5% and 35.08% of isolates, respectively (1). Liu et al. showed that all of the 85 *B. melitesis* isolates that were obtained from brucellosis patients in China were susceptible to levofloxacin, ciprofloxacin, sparfloxacin, minocycline, gentamicin tetracycline and doxycycline. Resistance to cotrimoxazole and rifampin was reported in 7.0% (6/85) and 1.0% (1.85) of the isolates, respectively (17). In another study, Abdel Maksoud and coworkers also reported high rates of resistance to rifampin in 64% of *Brucella* strains isolated from egyptian patients (13).

Similarly, Lopez-Merino et al. showed that cotrimoxazole and rifampin have a low inhibitory activity against *Brucella* strains (18). In an endemic area for human brucellosis in Turkey, *B. melitensis* isolates showed the highest resistance rate against cotrimoxazole (46.3%), whereas the resistance to rifampin has been only observed in 9.7% (19). In many developing countries, antibiotic resistance is the main result of inappropriate drug administration, which leads to the use of numerous antibiotics annually (20). It has also been reported that the consumption of systemic antibacterial agents such as broad-spectrum penicillins and third-generation cephalosporins and quinolones in Iran is much higher than other countries (20). Therefore, the present study evaluated a panel of seven antibiotics commonly used in the treatment of brucellosis. Rifampicin, also known as rifampin, is a commonly used antibiotic for the treatment of brucellosis, exerting its bactericidal activities by blocking the bacterial RNA and protein synthesis (21). This antibiotic also exhibits *in vitro* inhibitory effect against *Brucella* spp. because of its good intracellular diffusion (22). In the present study, 98.5% (59/60) of tested *Brucella* isolates with MIC≤1 μg/mL were considered sensitive to rifampin based on the CLSI breakpoints for slow-growing bacteria. Also, one of the *Brucella* strains was resistant to rifampin with a MIC of 1.5 μg/mL. High rate of resistance to rifampin has been previously reported in in egyptian field strains (64%) (13) as well as in Brazil (36.73%) (15), Turkey (9.7%) (19) and Malaysia (70%) (23).

It must be considered that a large number of brucellosis patients cannot tolerate prolonged therapy with rifampin because of its adverse gastrointestinal reactions. In this regard, the co-administration of streptomycin and doxycycline was reported to be the regimen of choice followed by the combination of rifampin and doxycycline as no therapeutic failures or relapse were reported following these regimens (24). However, numerous studies revealed that rifampin should be prescribed with caution, because of the elevated frequency of intermediate sensitivity to these drugs (1).

Our results also confirmed the observations of previous studies regarding the absence of resistance to streptomycin and doxycycline regimens among *Brucella* strains (17).

A mixture of trimethoprim and sulphamethoxazole has been suggested as an alternative antimicrobial agent for the brucellosis treatment in children and pregnant females (25). However, numerous studies reported that there is resistance to cotrimoxazole in *B. melitensis* (17, 18, 26). Our results showed an excellent activity of this drug against the Iranian *B. melitensis* isolates and no resistance to cotrimoxazole was observed among 54 *Brucella* strains.

In conclusion, most of the antibacterial drugs tested in this study, except ampicillin-sulbactam revealed an effective inhibitory activity against *B. melitens* and could be considered for therapeutic regimens. Appropriate cautions should be taken in the prescription of ampicillin-sulbactam and rifampin due to observed resistances. Finaly, the intracellular localization of *Brucella* restricts the selection of effective antimicrobial drugs against localized and systemic brucellosis.
